# Feto-placental endothelial cells of female neonates are more susceptible to gestational diabetes-induced changes

**DOI:** 10.1007/s00418-025-02433-x

**Published:** 2025-11-25

**Authors:** Silvija Tokic, Axel Schlagenhauf, Katrin A. Dohr, Gernot Desoye, Ursula Hiden

**Affiliations:** 1 Department of Paediatrics and Adolescent Medicine, Medical University of Graz, Auenbruggerplatz 34/2, 8036 Graz, Austria; 2 Research Unit Analytical Mass Spectrometry, Cell Biology and Biochemistry of Inborn Errors of Metabolism, Medical University of Graz, Graz, Austria; 3 Division of General Paediatrics, Department of Paediatrics and Adolescent Medicine, Medical University of Graz, Graz, Austria; 4 Department of Obstetrics and Gynecology, Medical University of Graz, Graz, Austria; 5 Research Unit Early Life Determinants, Medical University of Graz, Graz, Austria

**Keywords:** Fetal sex, Endothelial cell, Placenta, Fetal programming, Gestational diabetes mellitus

## Abstract

Fetal sex influences gene expression in the healthy feto-placental endothelium, potentially contributing to sex-dependent developmental programming and disease risk. Gestational diabetes mellitus (GDM) alters maternal–fetal homeostasis and placental vascular function. Building on previous findings of sex-biased gene expression in healthy feto-placental endothelial cells (fpEC), we investigated whether these biases persist or change following GDM exposure. We first identified sex-biased gene expression in fpEC from GDM pregnancies, then analyzed GDM-induced changes separately in male and female fpEC. Gene ontology enrichment was performed using the PANTHER database. Proliferation and network formation were assessed by BrdU incorporation assay and Matrigel assay, respectively. Female fpEC exhibited a greater transcriptional response to GDM, with more differentially expressed genes than male cells. Functionally, GDM reduced proliferation and increased network formation in female fpEC, while male cells were comparatively unaltered. In healthy conditions, male and female fpEC showed clear transcriptomic and functional dimorphism, which was abolished by GDM. Interestingly, GDM amplified sex-biased gene expression despite convergence in cellular behavior. These findings highlight fetal sex as a key modifier of the placental endothelial response to GDM and support its relevance in sex-specific pregnancy outcomes.

**Supplementary Information:**

The online version contains supplementary material available at 10.1007/s00418-025-02433-x.

## Introduction

Fetal sex is an important determinant of pregnancy physiology and outcome, particularly in the context of gestational diabetes mellitus (GDM). Meta-analyses and large cohort studies have shown that carrying a male fetus confers a higher maternal risk of developing GDM (Jaskolka et al. 2015; Maghalian et al. 2025), likely due to lower maternal β-cell function and elevated glucose levels in these pregnancies (Retnakaran et al. 2015). Once GDM is established, fetal sex also modulates perinatal outcomes: male fetuses are associated with higher rates of macrosomia, large-for-gestational-age birth, and obstetric complications such as cesarean delivery (Maghalian et al. 2025). These observations underscore a fundamental sex dimorphism in utero, wherein male and female fetuses respond differently to the metabolic challenges of pregnancy—a bias that may contribute to lifelong differences in disease susceptibility.

The placenta is considered a key mediator of these fetal sex differences. As both an endocrine organ and a site of nutrient and gas exchange, it plays a central role in supporting fetal growth through its vascular interface, the feto-placental endothelium (Burton and Fowden 2015). Despite similar size, placentas of male fetuses support higher fetal growth rates, suggesting greater nutrient transfer efficiency and sex-dependent stress responsiveness (Brett et al. 2014). In GDM, maternal and fetal hyperglycemia, coupled with altered hormone and growth factor levels, induce structural and functional remodeling of the placenta (Desoye and Hauguel-de Mouzon 2007; Hiden et al. 2012), including hypervascularization and increased villous branching (Jirkovská et al. 2002; Troncoso et al. 2017). These angiogenic adaptations are thought to compensate for increased fetal metabolic demands and are closely linked to GDM-associated complications (Cardenas et al. 2018; Wang et al. 2023). Importantly, many adverse outcomes, including increased insulin use (Giannubilo et al. 2018) and elevated maternal risk for postpartum type 2 diabetes (Broere-Brown et al. 2020), are more pronounced in male-bearing pregnancies.

The mechanisms underlying these sex-specific effects are actively being explored. While adult sex hormones influence endothelial function later in life (Senthil Kumar et al. 2023), sex chromosome composition and intrinsic gene regulation within the placenta contribute to sex-biased developmental trajectories from early gestation. Multiomic profiling has revealed widespread sex-specific gene expression across trophoblasts, immune cells, and endothelial cells in the human placenta. Female placentas often upregulate immune and growth-related pathways, while male placentas prioritize nutrient transport and display distinct immune profiles (Braun et al. 2022). Consistently, isolated feto-placental endothelial cells (fpEC) after healthy pregnancies show sex-specific transcriptional programs and phenotypes, including differences in cytoskeletal organization, barrier properties, and angiogenic signaling (Cvitic et al. 2013, 2020). GDM alters epigenetic and transcriptomic responses in fpEC with effects on barrier function (Cvitic et al. 2018) and more pronounced effects in fpEC from female fetuses (Strutz et al. 2018). These findings support the idea that male and female placentas adopt distinct adaptive strategies in response to diabetic pregnancy.

Here, we build on our previous findings of sex-biased gene expression and function in healthy fpEC to investigate whether these differences persist following GDM exposure. Specifically, we ask whether GDM disrupts or amplifies existing sex-biased transcriptional programs and functional phenotypes in fpEC, and if so, which sex is more affected. To address this, we compare male and female fpEC from healthy and GDM pregnancies using transcriptomic profiling and functional assays.

## Materials and methods

### Sample collection

Ethical approval was obtained from the Medical University of Graz (25-008ex12/13, 27-268ex14/15 and 29-319ex16/17) and the study was performed in accordance with the Declaration of Helsinki. Written informed consent was obtained from all participants. Control placentas were collected from pregnancies of non-smoking (self-reported) women with a negative 75 mg oral glucose tolerance test (oGTT) performed at 24–28 weeks of gestation and who were free of medical disorders or pregnancy complications such as pre-eclampsia and fetal growth restriction. GDM was diagnosed according to WHO/IADPSG criteria (International Association of Diabetes and Pregnancy Study Groups Consensus Panel et al. 2010). Women with positive oGTT were either recommended a diet or were additionally treated with insulin (NovoRapid plus Insulatard; NovoNordisk Pharma, Vienna, Austria).

### Subjects’ characteristics

Maternal, fetal, and placental characteristics of the cohorts used for proliferation and 2D network formation assay are shown in Table 1. Statistical comparison was performed for male and female neonates within the healthy and GDM group, as well as comparison of GDM impact on male and female neonates separately. In our cohort, neonatal length was significantly increased in healthy male neonates compared to healthy female neonates, which is in line with previous reports (Galjaard et al. 2019). All other parameters were independent of fetal sex.

**Table 1:** Maternal, neonatal, and placental clinical characteristics

	Male	Female	GDM male	GDM female
Neonatal data				
Number of individuals used for isolation	9	11	6	5
Offspring weight (g)	3485.5 ± 323.7	3343.1 ± 409.5	3565.0 ± 345.6	3324.0 ± 185.1
Offspring length (cm)	51.6 ± 1.9	49.7 ± 1.5*	51.3 ± 1.2	50.0 ± 2.8
Placental weight (g)	576.7 ± 73.0	649.5 ± 191.6	662.0 ± 184.5	640.0 ± 131.0
C-peptide (nmol/l)	ND	ND	10.71 ± 4.30	12.83 ± 7.88
Insulin (pmol/l)	ND	ND	51.30 ± 1.25	50.00 ± 2.83
Maternal data				
Gestational age (weeks)	39.4 ± 0.8	39.3 ± 1.1	39.4 ± 0.8	39.3 ± 0.8
Mode of delivery (vaginal/C-section)	5/4	6/5	3/3	2/3
Age (years)	33.6 ± 5.7	28.6 ± 7.5	29.7 ± 6.9	30.0 ± 2.9
Height (m)	1.65 ± 0.06	1.70 ± 0.05	1.64 ± 0.01	1.63 ± 0.09
Weight before pregnancy (kg)	59.6 ± 8.8	72.0 ± 17.3	73.0 ± 17.7	67.5 ± 22.8
BMI before pregnancy	21.8 ± 2.7	24.5 ± 5.2	27.1 ± 6.3	25.2 ± 7.9
Weight before birth (kg)	73.5 ± 11.3	91.1 ± 13.3	85.4 ± 21.2	86.5 ± 21.8
BMI before birth	26.2 ± 3.6	32.7 ± 6.4	31.7 ± 7.6	32.2 ± 7.5
CRP (nmol/l)	8.53 ± 5.30	7.30 ± 5.70	2.98 ± 1.76	6.98 ± 3.32
Cell culture data				
Cell isolation passage	8.25 ± 0.83	6.71 ± 1.28	7.60 ± 0.80	6.50 ± 0.87

Data are given as mean ± SD

*ND* not determined

*Indicates significance level *p* < 0.05 for comparison of male and female healthy neonates

### Cell culture

Primary arterial fpEC were isolated from third trimester human placentas after healthy pregnancies following a standard protocol (Lang et al. 2008). Cells were characterized by immunocytochemical analysis as described previously (Lang et al. 2003). GDM and control fpEC were separately cultured on 1% (v/v) gelatin-coated (Sigma-Aldrich, Merck KGaA, Darmstadt, Germany) flasks (75 cm^2^, Corning, NY, USA) using Endothelial Basal Medium (EBM, Cambrex, Clonetics, USA) supplemented with the EGM-MV BulletKit (Cambrex, Clonetics, USA) in normoglycemic conditions (5.5 mM glucose).

### Data assessment, analysis, and gene ontology

This study used normalized and log-transformed previously published gene expression data (Cvitic et al. 2018) available on the Gene Expression Omnibus (GEO) database (GSE 103552). To assess sex differences in gene expression specifically within GDM pregnancies, a one-way analysis of variance (ANOVA) was performed using fetal sex as the independent factor. This analysis included only samples from GDM pregnancies and was applied gene-wise across the dataset.

To assess GDM effects within each sex, the dataset was split into male and female groups. One-way ANOVA was then performed separately within each group using GDM status as the independent factor. This stratified analysis enabled identification of genes differentially expressed in response to GDM in male or female fpEC. The *p* values were adjusted for multiple testing using the Benjamini–Hochberg method but none of the genes reached significance. Genes with an unadjusted *p* value < 0.05 and an absolute fold-change (FC) ≥ 1.3 were used for gene ontology analysis using PANTHER (version 19.0) database (Thomas et al. 2022).

### Bromodeoxyuridine (BrdU) cell proliferation ELISA assay

Determination of proliferation was performed by measuring bromodeoxyuridine (BrdU) incorporation into DNA using enzyme-linked immunosorbent assay (ELISA) (Kohara et al. 2020). The assay was performed according to the manufacturer’s instructions (CycLex BrdU ELISA Kit, CycLex Co., Ltd, Nagano, Japan) by seeding 5000 cells per well in a 96-well microplate in a final volume of 100 μl of supplemented EBM with 5% FCS. After culture for 48 h at 21% O_2_ and 37 °C, BrdU was added to a final concentration of 10 μM and incubated for 2 h at 37 °C. The medium was removed and cells were fixed and denaturized using the fixing/denaturing solution provided by the manufacturer within 30 min incubation at room temperature. Fixing was followed by 1 h incubation with primary anti-BrdU monoclonal antibody and then 1 h incubation with HRP-conjugated anti-mouse IgG secondary antibody and with washing steps in between. Substrate reagent (50 μl) was added and after 15 min incubation the stop solution was added. Absorbance was measured immediately at 450/540 nm on a FluoSTAR Optima 413 spectrofluorimeter (BMG Labtechnologies, Offenburg, Germany).

### In vitro 2D-network formation assay on Matrigel

In vitro tube formation assay was performed in 96-well plates coated with 50 µl of growth factor reduced Matrigel basement membrane matrix (BD Biosciences, Franklin Lakes, NJ, USA). After polymerization (30 min at 37 °C) fpEC (8000 cells/well) were plated in triplicates. Male and female control (*n* = 8; m/f = 4/4) and GDM (*n* = 8; m/f = 4/4) fpEC isolations were used to compare the effect of fetal sex. The cells were used between passages 5 and 9. Network formation was monitored at 21% O_2_ for 24 h and images were taken at 3, 6, 12 and 24 h using a digital camera attached to an inverted phase-contrast microscope (Carl Zeiss AG, Oberkochen, Germany) equipped with a ×10 Plan-Apochromat objective (N.A. 0.25). Nine adjacent fields per well were captured and stitched using ZEN imaging software (Carl Zeiss AG). Quantification was performed by measuring the total tube length using AngioJ-Matrigel assay plugin for the ImageJ software (NIH) (Guidolin et al. 2004). The plugin was developed and kindly provided by Diego Guidolin, Department of Human Anatomy and Physiology, Section of Anatomy, University of Padova, Padova, Italy.

### Statistical analysis

To identify differences in the subjects’ characteristics between male and female neonates, Student’s *t* test was used after testing for normal distribution. Proliferation and network formation data were analyzed using two-way ANOVA with fetal sex and disease as fixed factors and Bonferroni as post hoc.

## Results

### Sex-biased gene expression is increased in fpEC after GDM pregnancies

**Fig. 1:**
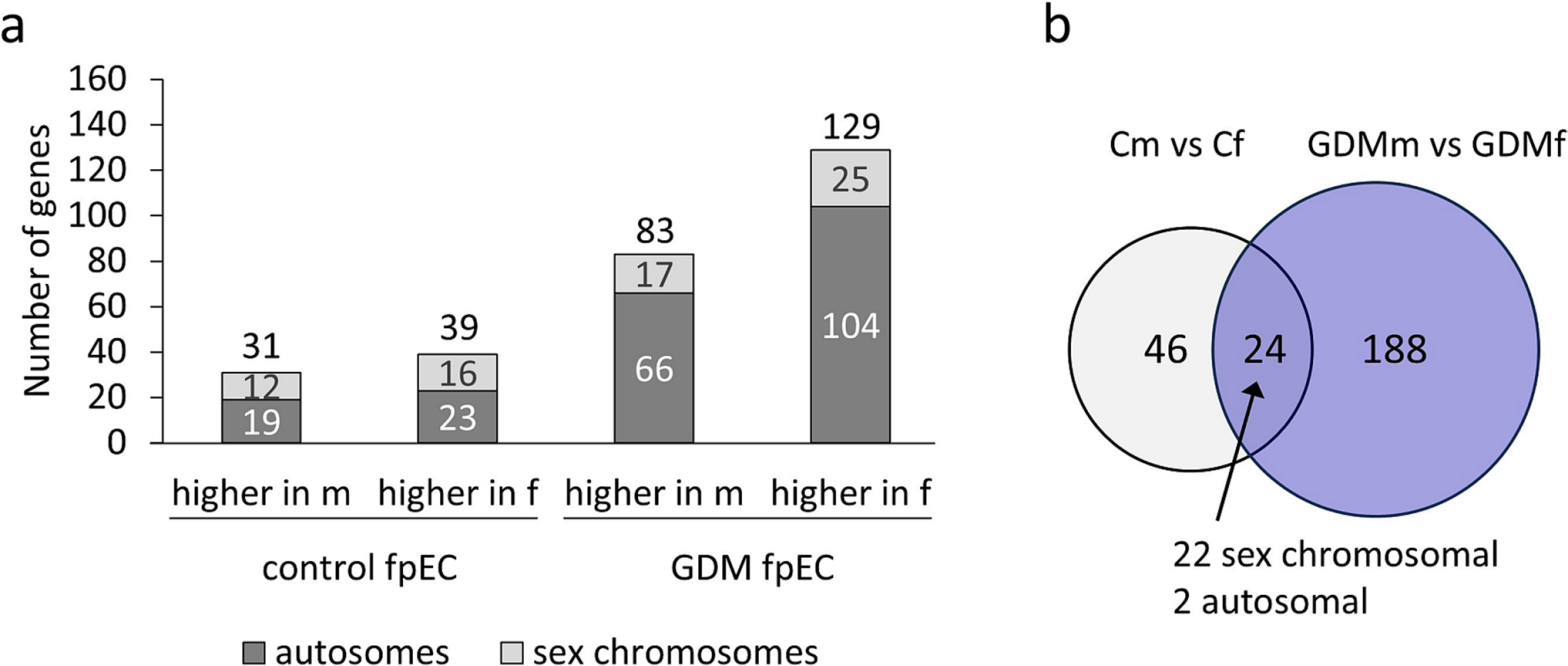
Genes with sex-biased expression in healthy versus GDM pregnancies. **a** Number of sex chromosomal and autosomal genes differentially expressed between male vs female fpEC after healthy and GDM pregnancies. The number above each column represents the total number of differentially expressed genes. **b** Venn diagram showing overlapping genes with sex-biased expression between healthy and GDM pregnancies. Microarray data for GDM fpEC were obtained from Cvitic et al. (2018) and analyzed after stratification by fetal sex. Differential expression was defined as *p* < 0.05 and fold change ≥ 1.3. Sample size: fpEC from female/male healthy pregnancy, *n* = 4/4; fpEC from female/male GDM pregnancy, *n* = 7/4. *Cm* male fpEC from control pregnancies, *Cf* female fpEC from control pregnancies, *GDMm* male fpEC from GDM pregnancies, *GDMf* female fpEC from GDM pregnancies

We previously identified 70 genes that were differentially expressed between male and female fpEC after a normal pregnancy (31 genes had higher expression in male fpEC, and 39 genes in female fpEC) (Cvitic et al. 2013). In this study, we firstly aimed to assess whether sex differences persist also in fpEC after GDM exposure. Hence, we re-evaluated microarray data from a previous study (Cvitic et al. 2018) (available at GEO, GSE 103552) and performed male vs female comparison in GDM samples. We identified 212 sex-biased genes (*p* < 0.05 and fold change ≥ 1.3, Fig. 1a), out of which 83 genes had higher expression in male fpEC, and 129 genes in female fpEC. In both healthy and GDM pregnancies, genes with sex-biased expression were distributed across autosomal and sex chromosomes (Fig. 1b). In total, 24 genes had stable sex-biased expression in healthy and GDM pregnancies, with 22 located on sex chromosomes and only two on autosomes (Fig. 1b). Ten of the 22 genes located on the sex choromosomes were on the Y and 12 on the X chromosome (Supplementary Tables 1 and 2).

### Biological functions of sex-biased genes after healthy and after GDM pregnancies

**Fig. 2:**
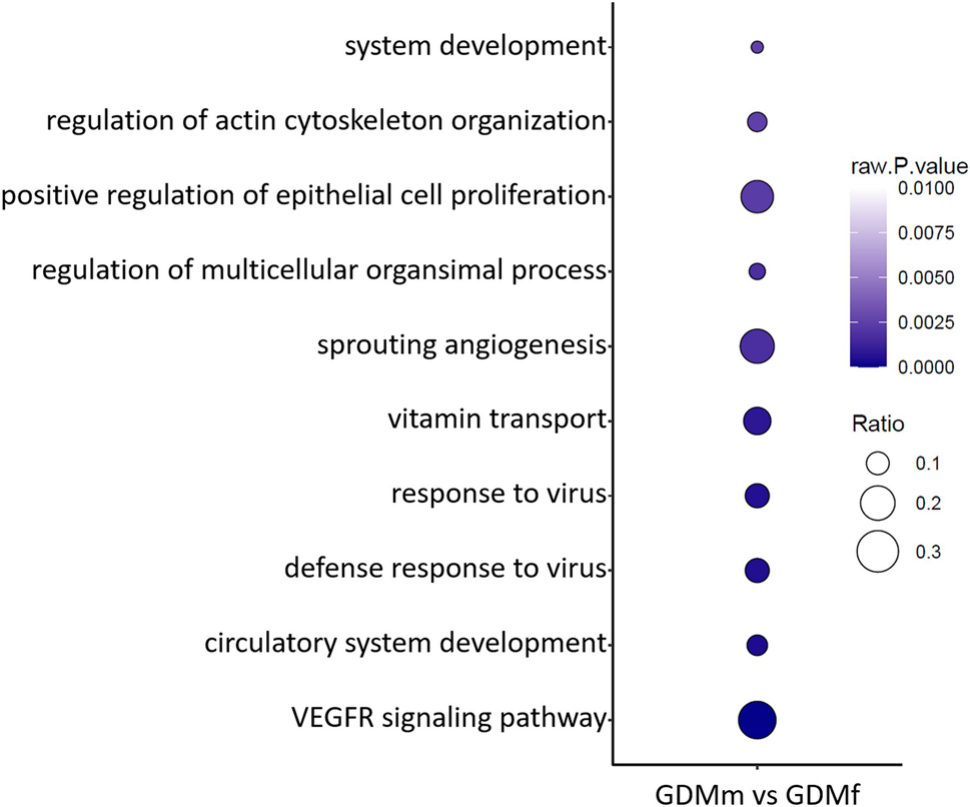
Biological processes affected by sex-biased genes in fpEC after GDM pregnancy. Pathway analysis was performed using PANTHER 19.0 analysis tool. *GDMm* male fpEC from GDM pregnancies, *GDMf* female fpEC from GDM pregnancies

To predict biological functions affected by differentially expressed genes in male versus female fpEC after GDM exposure, we performed functional annotation using the established online tool PANTHER (Thomas et al. 2022). The list of sex-biased genes after healthy pregnancies was annotated using GeneCards database (https://www.genecards.org). Although the number of sex-biased genes in fpEC differs more than twofold between healthy and GDM pregnancies (Fig. 2), the affected pathways are very similar and primarily include pathways related to immune function, as well as pathways associated with migration, actin organization, proliferation, and angiogenesis (Fig. 2, Supplementary Table 2).

### GDM alters more genes in female than male fpEC

As GDM seemed to increase sex biased gene expression, we next aimed to determine if male and female fpEC are equally affected by GDM. To answer this question, we segregated data of the GSE 103552 dataset according to fetal sex and compared the effect of GDM on gene expression separately in male and female samples.

**Fig. 3:**
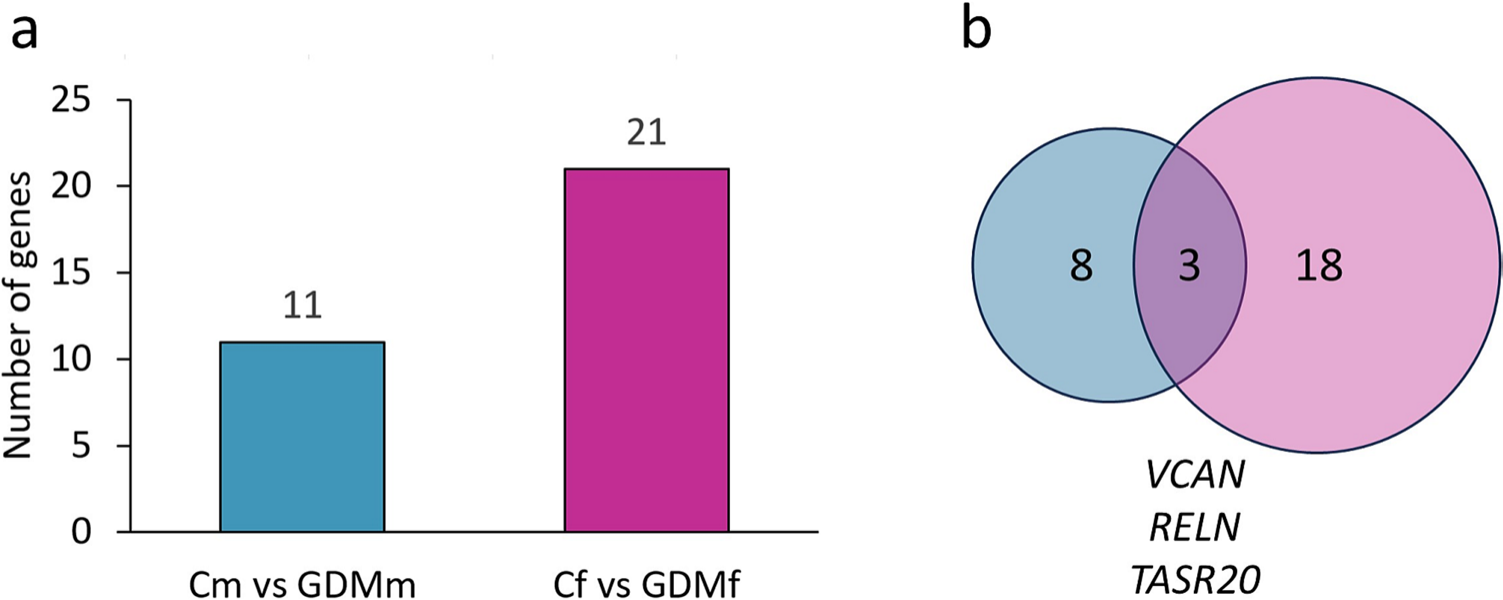
Sex-dependent gene expression in fpEC after GDM exposure. **a** Number of genes differentially expressed in fpEC from male and female fetuses after GDM exposure. **b** Overlap of genes altered by GDM in male and female fpEC. Microarray data was used from 15 and reanalyzed after stratification by fetal sex. Genes were considered differentially expressed with a fold change > 1.3 and *p* < 0.05. Sample size: male fpEC from healthy/GDM pregnancy, *n* = 4/4; female fpEC from healthy/GDM pregnancy, *n* = 4/7. *Cm* male fpEC after normal pregnancies, *GDMm* male fpEC after GDM pregnancies, *Cf* female fpEC after normal pregnancies, *GDMf* female fpEC after GDM pregnancies

GDM altered the expression of 11 genes in male fpEC vs 21 genes in female fpEC (Fig. 3a), with three genes affected by GDM in fpEC of both sexes (Fig. 3b). Thus, 26 genes were affected by GDM specifically linked to male or female sex. Most of these genes were upregulated by GDM, specifically 10 in male vs 14 in female cells. Three genes, *VCAN* (versican), *RELN* (reelin), and *TAS2R20* (taste 2 receptor member 20), were upregulated in both male and female fpEC (Table 2).

**Table 2:** Genes altered by GDM in male and female fpEC

Male fpEC: C vs GDM				
Gene	Description (function)	Chromosome	*p* value	FC
TPTE	Transmembrane phosphatase with tensin homology (signal transduction pathways)	chr21	0.021	1.57
**VCAN**^cc,m^	Versican (cell adhesion, proliferation, migration, angiogenesis)	chr5	0.010	1.51
ZFP57	Zinc finger protein 57 (transcription regulator, maintenance of maternal and paternal gene imprinting)	chr6	0.046	1.42
FAP^cc,m^	Fibroblast activation protein α (extracellular matrix degradation, tissue remodelling, proliferation)	chr2	0.038	1.42
GPR112	Adhesion G protein-coupled receptor G4 (DGRG4) (transmembrane signaling receptor)	chrX	0.004	1.37
**RELN**^m^	Reelin (control of cell–cell interactions, migration)	chr7	0.007	1.37
ERAP2^i^	Endoplasmic reticulum aminopeptidase 2 (peptide trimming for the generation of HLA class I-binding peptides)	chr5	0.011	1.36
CCDC144A	Coiled-coil domain containing 144A (calcium oxalate binding activity)	chr17	0.025	1.35
NCAM2^m^	Neural cell adhesion molecule 2 (cell–cell interaction)	chr21	0.003	1.34
**TAS2R20**^i^	Taste 2 receptor member 20 (bitter taste perception, innate immunity)	chr12	0.004	1.31
HLA-DPB1^i^	Major histocompatibility complex, class II, DP β1 (antigen presentation)	chr6	0.029	− 1.31

Female fpEC: C vs GDM				
Gene	Description (function)	Chromosome	*p* value	FC
**VCAN**^cc,m^	Versican (cell adhesion, proliferation, migration, angiogenesis)	chr5	0.000	1.74
POSTN^cc,m^	Periostin (ECM protein, support of adhesion, migration, and proliferation)	chr13	0.012	1.51
IFNE^i^	Interferon ε (immune response)	chr9	0.000	1.40
BLID^cc^	BH3-like motif containing, cell death inducer (proapoptotic)	chr11	0.000	1.37
TAS2R4^i^	Taste 2 receptor member 4 (bitter taste perception, innate immunity)	chr7	0.001	1.37
**TAS2R20**^i^	Taste 2 receptor member 20 (bitter taste perception, innate immunity)	chr12	0.000	1.37
TAS2R31^i^	Taste 2 receptor member 31 (bitter taste perception, innate immunity)	chr12	0.000	1.37
KCNMA1^i^	Potassium calcium-activated channel subfamily M α1 (export of K^+^, contraction of smooth muscle, innate immunity)	chr10	0.000	1.35
KCNJ6	Potassium inwardly rectifying channel subfamily J member 6 (potassium flow into the cell)	chr21	0.016	1.35
IGFBP5^cc^	Insulin like growth factor binding protein 5 (regulating the availability of IGFs to their receptors, stimulates proliferation)	chr2	0.045	1.34
TAS2R19^i^	Taste 2 receptor member 19 (bitter taste perception, innate immunity)	chr12	0.000	1.33
DOCK5^m^	Dedicator of cytokinesis 5 (guanine nucleotide exchange factor (GEF) for Rho and Rac; endothelial cell spreading and migration)	chr8	0.004	1.32
GPR21	G protein-coupled receptor 21 (signal transduction pathway)	chr9	0.000	1.31
**RELN**^m^	Reelin (control of cell–cell interactions, migration)	chr7	0.002	1.31
TSPAN7^cc,m^	Tetraspanin 7 (signal transduction events in the regulation of proliferation and cell motility)	chrX	0.008	− 1.32
ART4	ADP-ribosyltransferase 4 (inactive) (Dombrock blood group)	chr12	0.009	− 1.32
MRAP2	Melanocortin 2 receptor accessory protein 2 (modulation of melanocortin receptor signaling)	chr6	0.000	− 1.33
TMEM71	Transmembrane protein 71	chr8	0.003	− 1.35
PLA2G4A^i^	Phospholipase A2 group IVA (membrane lipid remodelling, biosynthesis of lipid mediators of the inflammatory response)	chr1	0.000	− 1.38
TSPAN8^cc,m^	Tetraspanin 8 (signal transduction events in the regulation of proliferation and cell motility)	chr12	0.028	− 1.50
MAEL^cc^	Maelstrom spermatogenic transposon silencer (intrinsic apoptotic signaling in response to DNA damage)	chr1	0.010	− 1.58

Genes differentially expressed between GDM vs control male and female fpEC, respectively. Microarray data were taken from Cvitic et al. (2018) and reanalyzed stratified by fetal sex. Differential expression was defined as fold change > 1.3 and *p* < 0.05. Gene functions were obtained from the GeneCards database (https://www.genecards.org). Sample size: male fpEC from healthy/GDM pregnancy, *n* = 4/4; female fpEC from healthy/GDM pregnancy, *n* = 4/7 . Genes in bold refer to genes deregulated by GDM independent of fetal sex.

*FC* fold-change (GDM vs control) within the male and the female group, respectively

^i^Genes related to immune processes

^cc^Genes related to cell cycle

^m^Genes related to cell–cell contacts, actin cytoskeleton, or migration

### GDM decreases proliferation of fpEC from female fetuses

*VCAN* and *RELN* are genes encoding ECM proteins known to regulate cell proliferation and migration (Islam and Watanabe 2020; Alexander et al. 2023). These and other genes are altered by GDM specifically in male fpEC (e.g., *FAP*, fibroblast activation protein α) (Oguri et al. 2024) and specifically in female fpEC (e.g., *POSTN*, periostin) (Nie et al. 2020). To determine whether these gene expression changes translate into functional alterations of fpEC, we investigated migration and cell cycle-related processes in vitro, specifically proliferation and network formation, in fpEC derived from male and female fetuses after healthy versus GDM pregnancies.

**Fig. 4:**
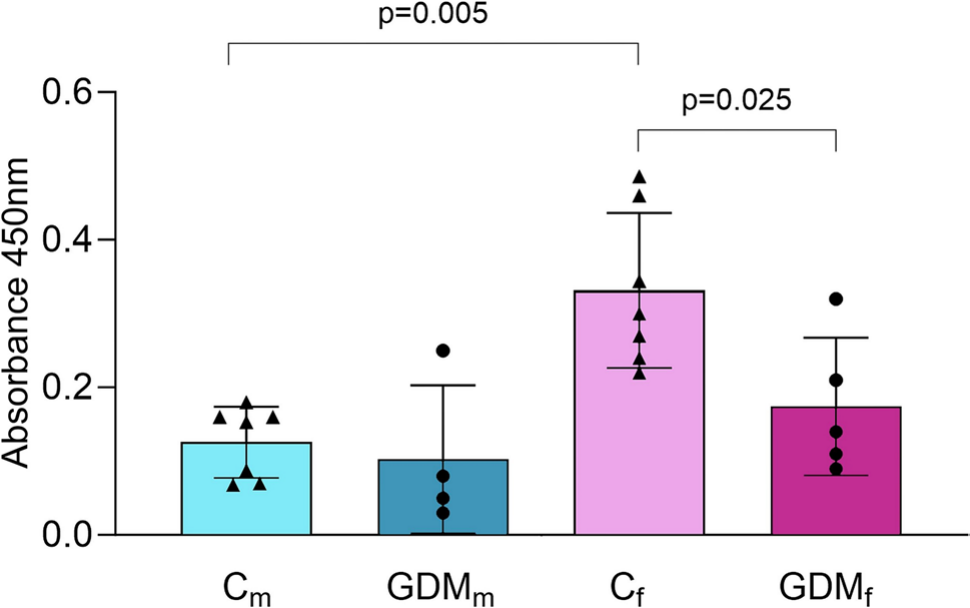
GDM reduces proliferation in female, but not male fpEC. BrdU incorporation in fpEC isolated from male and female fpEC after healthy and GDM pregnancies. Statistical analysis was performed using two-way ANOVA with GDM and fetal sex as fixed factors. A *p* value < 0.05 was considered statistically significant. Sample size: male fpEC from healthy/GDM pregnancy, *n* = 7/4; female fpEC from healthy/GDM pregnancy, *n* = 7/5. Data are presented as mean ± SD from individual fpEC isolations, each measured in triplicate. *Cm* male fpEC after normal pregnancies, *GDMm* male fpEC after GDM pregnancies, *Cf* female fpEC after normal pregnancies, *GDMf* female fpEC after GDM pregnancies

Proliferation was assessed using a BrdU ELISA, which measures incorporation of BrdU into newly synthesized DNA during the S-phase of the cell cycle. GDM exposure had no significant effect on BrdU incorporation in male fpEC, but it significantly reduced proliferation in female fpEC (− 47%, *p* = 0.025) (Fig. 4). After healthy pregnancy, female fpEC showed significantly higher BrdU incorporation (+ 62%; *p* = 0.0005) compared to male cells, a sex-specific difference that was absent following GDM exposure.

### GDM increases network formation of fpEC from female fetuses

Since GDM also affected the expression of genes involved in cell migration (Table 2), we assessed in vitro network formation as a surrogate marker of cell motility. Male and female fpEC isolated after healthy or GDM pregnancies were seeded on Matrigel and analyzed over 24 h.

**Fig. 5:**
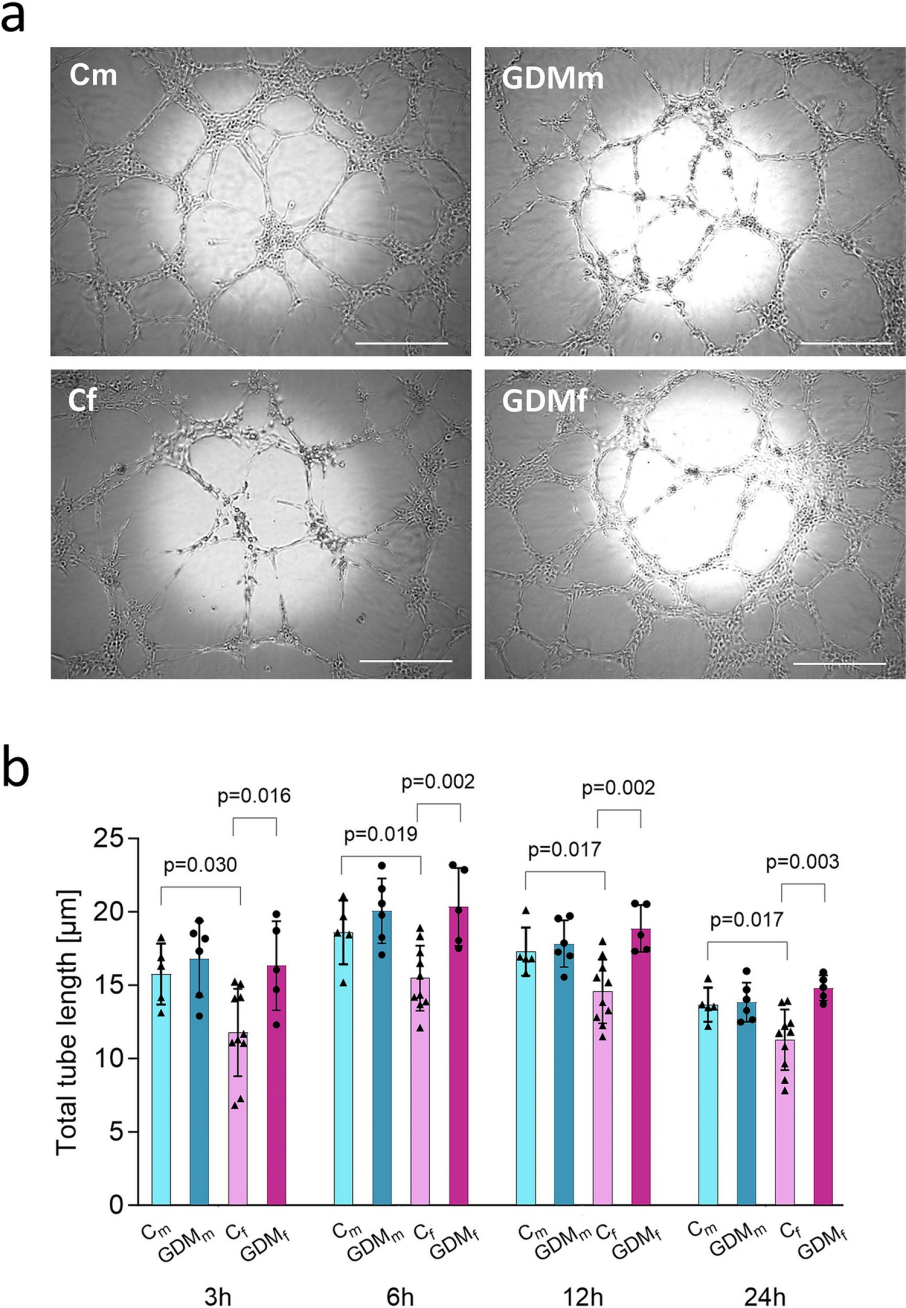
GDM increases network formation in female, but not male fpEC **a** Representative images of network formation on Matrigel of male and female fpEC after healthy and GDM pregnancies over the course of 24 h. Scale bar is 200 µM **b** Quantification of network formation (total tube length) using ImageJ. Statistical analysis was performed separately for each time point using two-way ANOVA with GDM exposure and fetal sex as fixed factors. A *p* value < 0.05 was considered statistically significant. Sample size: male fpEC from healthy/GDM pregnancy, *n* = 6/6; female fpEC from healthy/GDM pregnancy, *n* = 10/5. Data are presented as mean ± SD from individual fpEC isolations, each measured in triplicate. *Cm* male fpEC after normal pregnancies, *GDMm* male fpEC after GDM pregnancies, *Cf* female fpEC after normal pregnancies, *GDMf* female fpEC after GDM pregnancies

In male fpEC, GDM had no significant effect on network formation. In contrast, female fpEC showed significantly increased network formation following GDM exposure at 3 h (+ 28% *p* = 0.016), 6 h (+ 24%, *p* = 0.002), 12 h (+ 23%, *p* = 0.002), and 24 h (+ 24%, *p* = 0.003). After healthy pregnancy, female fpEC formed significantly shorter networks than male fpEC at all time points: 3 h (− 23%, *p* = 0.03), 6 h (− 16%, *p* = 0.019), 12 h (− 16%, *p* = 0.017), and 24 h (− 17%, *p* = 0.027). These sex-specific differences were no longer observed in fpEC from GDM pregnancies (Fig. 5a, b).

## Discussion

Pregnancy complications such as GDM have been shown to cause distinct clinical outcomes favoring female offspring (Li et al. 2017). However, research investigating sex-dependent effects, particularly in relation to endothelial function, remains limited. We have previously demonstrated that GDM affects miRNA expression in fpEC in a sex-dependent manner (Strutz et al. 2018), but functional consequences of this regulation were not established. In the present study, we extend our earlier work by exploring sex-biased gene expression in fpEC after GDM pregnancy and assess whether these transcriptomic differences translate into sex-specific functional alterations. Our findings demonstrate that (i) female fpEC exhibited a greater transcriptional and functional response to GDM, potentially indicating adaptability towards GDM environment; (ii) under healthy conditions, male and female fpEC displayed transcriptomic and functional sex-dimorphism in terms of proliferation and network formation; (iii) GDM exposure abolished the functional sex-dimorphism but amplified sex-biased gene expression in fpEC.

(i) Female fpEC showed changed expression level in almost twice as many genes as their male counterparts after GDM, namely 21 vs 11. Specifically, GDM upregulated two genes in male fpEC that were previously linked to other pregnancy disorders, *TPTE* and *NCAM2*, respectively. *TPTE* encodes a transmembrane phosphatase with tensin homology and was reported altered in early- and late-onset of pre-eclampsia (Weitzner et al. 2020), whereas *NCAM2*, a neural cell adhesion molecule, was upregulated in placentas after a preterm labor. Similar to our results, upregulated *NCAM2* was specific for male fetuses (Akram et al. 2022). These results suggest that in male fpEC GDM altered expression of genes that are sensitive to regulation by distinct intrauterine insults and, hence, are rather unspecific for GDM. In contrast, in female fpEC, GDM induced upregulation of several bitter taste receptor genes (*TAS2R4*, *TAS2R19*, *TAS2R20*, *TAS2R31*), which are extra-orally expressed GPCRs implicated in placental immune modulation (Taher et al. 2019) and may represent an adaptive response to GDM-associated inflammation.

Besides genes related to immune function, among the 26 genes differentially expressed between male and female fpEC by GDM, 12 were involved in migration and cell cycle, frequently with roles in both processes, e.g., *FAP* (fibroblast activation protein α), *TSPAN7* (tetraspanin 7), and *TSPAN8* (tetraspanin 8). Independent of fetal sex, GDM upregulated expression of ECM protein-encoding genes *VCAN* and *RELN*, important regulators of cell proliferation and migration. In line with the transcriptional data, GDM significantly reduced proliferation and increased network formation in female fpEC only, suggesting a shift toward a more migratory, activated endothelial phenotype (Carmeliet and Jain 2011; Rosano et al. 2020). This functional phenotype is consistent with the upregulation of immune-related genes and mirrors our previous findings that GDM affects a greater number of miRNAs in female fpEC (Strutz et al. 2018). Moreover, female placentas are known to exhibit greater immune adaptability during maternal stress (Baines and West 2023; Clifton 2010), which may contribute to the heightened transcriptional and functional responsiveness observed in female fpEC.

(ii) In healthy pregnancies, fpEC exhibit a sex bias in gene expression, proliferation, and network formation. We and others have previously reported sex differences in feto-placental and umbilical cord endothelial cells on gene expression and functional level. For instance, we identified differences in miRNA expression profiles, in vitro barrier function, and actin organization (Cvitic et al. 2020). Furthermore, applying the same analytical criteria used here (i.e., unadjusted *p* value, FC ≥ 1.3) in a combined analysis of arterial and venous placental endothelial cells, collectively referred to as “placental endothelium”, and using PANTHER pathway analysis tool, we identified “response to stimulus” and “immune system process” as key functional pathways showing sex-biased gene expression (Cvitic et al. 2013).
(iii) GDM altered the sex bias in gene expression and function observed in fpEC from healthy pregnancies. GDM increased the number of genes differentially expressed between male and female fpEC. This intensified sex-bias in gene expression after exposure to GDM is not surprising, as hyperglycemia (Gaspar et al. 2024), hyperinsulinemia (Arcaro et al. 2002; Watson et al. 2022), and inflammation (Mackay et al. 1993; Huang and Vita 2006) as main drivers of GDM environment have well-known effects on endothelium. Gene ontology analysis by the PANTHER software tool indicated that the genes characterized by a sex bias in GDM were involved in proliferation, angiogenesis, and immune system-related processes. These pathways were among the most frequent in the male–female differentially expressed genes after healthy pregnancies, even though the number of sex-biased genes more than doubled in GDM compared to healthy pregnancies. We believe this indicates that angiogenesis, growth, and immunity-related processes represent stable pathways with inherent sex differences in fpEC.

Consistent with previous studies, most sex-biased genes in fpEC were autosomal rather than sex-linked (Sood et al. 2006), suggesting that transcriptional sex differences extend beyond sex chromosome dosage. Several studies have shown female-biased upregulation of autosomal genes in the placenta, potentially reflecting enhanced adaptability to stressors such as infection or inflammation (Mao et al. 2010; Kassam et al. 2019). Similarly, female endothelial cells (e.g., HUVECs) have demonstrated greater resilience to metabolic and angiogenic stress (Lorenz et al. 2019). In our study, although GDM amplified transcriptomic sex differences, these did not translate into exaggerated functional differences. Instead, functional dimorphism observed in healthy fpEC was abolished in GDM, possibly as a result of compensatory mechanisms that buffer functional output despite divergent transcriptional programs. Interestingly, the 12 genes showing stable sex-biased expression across both healthy and GDM conditions were primarily X-linked, suggesting that these may underlie inherent and persistent differences in endothelial cell function between sexes.

A limitation of our study is the small sample size, which reduced statistical power for differential gene expression analysis following multiple testing correction reflecting other omics studies with small sample size (Ruchat et al. 2013; Rosario et al. 2021). Nevertheless, functional validation supports the biological relevance of the observed transcriptomic changes. Stratification by fetal sex revealed sex-specific responses that would otherwise remain undetected.

In conclusion, female fpEC showed greater transcriptional responsiveness and altered function in response to GDM, potentially indicating enhanced adaptability to metabolic stress. Further studies are needed to validate these findings and clarify why male fetuses appear more vulnerable to GDM-associated complications despite a more modest endothelial response. This work reinforces the importance of considering fetal sex in studies of placental function and developmental programming.

## Supplementary Information

Below is the link to the electronic supplementary material.

Supplementary file1 (XLSX 26 KB)

Supplementary file2 (XLSX 15 KB)

## Data Availability

All data supporting the results are presented in the manuscript. Transcriptomic data are available at the GEO database under the entry GSE 103552.
